# Perioperative systemic treatments in renal cell carcinoma

**DOI:** 10.3389/fonc.2024.1362172

**Published:** 2024-05-21

**Authors:** Rohit Goswamy, Ecem Kalemoglu, Viraj Master, Mehmet Asim Bilen

**Affiliations:** ^1^ Department of Hematology and Medical Oncology, Emory University School of Medicine, Atlanta, GA, United States; ^2^ Department of Hematology and Medical Oncology, Winship Cancer Institute of Emory University, Atlanta, GA, United States; ^3^ Department of Biochemistry, Emory University School of Medicine, Atlanta, GA, United States; ^4^ Department of Urology, Emory University School of Medicine, Atlanta, GA, United States

**Keywords:** renal cell carcinoma, neoadjuvant, adjuvant, kidney cancer, review, cancer, RCC (Renal Cell Carcinoma), outcomes

## Abstract

In this review, we aim to provide a comprehensive assessment of the evolving landscape of the perioperative management in renal cell carcinoma (RCC), emphasizing its dynamic and intricate nature. We explore academic and clinical insights into the perioperative treatment paradigm of RCC. Up-to-date treatment options are discussed and the evolving role of neoadjuvant and adjuvant therapy in RCC is highlighted.

## Introduction

In the U.S., there are 82,000 new renal cell carcinoma (RCC) cases and 15,000 deaths annually (1). Globally, RCC is estimated to cause 400,000 new cases and 170,000 deaths (2). In recent years, the treatment strategies in the localized and metastatic stages of RCC have changed dramatically. Correspondingly, the outcomes have improved substantially and patients with RCC are living longer as a result of these therapeutic advances. The five-year survival rate of patients with RCC has more than doubled over the last 50 years (3). Additionally, the incidence of RCC has now risen more than threefold compared to the mortality rate, suggesting improved survival and a lower case fatality rate. This is likely due to earlier detection and advancements in surgical outcomes and therapeutics (1, 4).

The treatment of RCC is a dynamic field, and strategies have changed considerably since the implementation of targeted therapies. The treatment plans are typically made based on the extent of the disease, the patient’s age, and comorbidities. The definitive strategy for patients with early-stage (I – III) RCC is curative intent surgery.

Unfortunately, many patients with RCC remain clinically asymptomatic during the course of the disease. As a result, these patients present with metastatic disease. However, because of the new therapeutic options, these patients have several treatment alternatives available to them including antiangiogenic therapy (vascular endothelial growth factor inhibitors [VEGFi]), mTOR (mechanistic target of rapamycin) inhibitors, checkpoint inhibitor immunotherapies (IO), and others. The outcomes in the metastatic setting have improved dramatically with the utilization of these therapies in isolation or combination. These targeted therapies and IO have changed the survival and quality of life outcomes for patients with advanced stage disease. Many of these patients now have long-lasting responses and are living years with improved quality of life. The successful results from the new therapies for metastatic cases have naturally raised questions about the application of these therapies in earlier stages of the disease process as well.

The use of these systemic therapies in the perioperative management of RCC is a complex and evolving landscape. The common theme is the repurposing of these proven metastatic stage, systemic agents in the localized, early stages of the disease. In the setting of locally advanced disease, some of these therapies normally reserved for the metastatic process have shown benefit as potential neoadjuvant therapies to permit surgical resection in those who otherwise would not have been deemed surgical candidates. This potentially allows for improved objective outcomes and long-term survival benefits. Additionally, many of these therapies have been evaluated in the adjuvant, post-nephrectomy arena to reduce the risk of recurrence, improve disease- free survival, and, ultimately, to improve overall survival outcomes.

Surgery plays an important role, even in the metastatic RCC setting. Surgical resection may be combined with systemic therapy as a combined modality approach to decrease tumor burden prior to systemic therapy or it may be used to remove the residual tumor after significant response to initial, upfront systemic therapy. Additionally, metastasectomy may also be used in selected patients with limited disease burden and metastatic deposits. Finally, as a palliative measure, surgery may be performed for severe, local symptoms directly resulting from the anatomical aberrations of the primary tumor.

Fundamentally, this review will evaluate currently available treatment options in the perioperative RCC landscape. Neoadjuvant therapies will be reviewed in two particular patient populations – those without evidence of metastatic disease (M0) in whom surgical resection is curative in nature and those with metastatic disease (M1) who receive pre-operative therapy to allow for cytoreductive nephrectomy in the setting of distant metastasis. The adjuvant therapy studies usually are more traditional in nature and include those therapies that are given after surgical intervention (as discussed later). The goals of neoadjuvant and adjuvant therapy approaches are similar – to improve disease-free survival (DFS) and overall survival (OS). However, neoadjuvant therapy also aims to achieve specific outcomes such as downsizing tumors, decreasing tumor thrombus burden, allowing for nephron-sparing surgery, and improving surgical outcomes. It should be noted that neoadjuvant therapy describes those patients with M0 disease who receive systemic therapy to allow for curative-intent surgical resection while those with M1 disease are more accurately thought of as “pseudoneoadjuvant” since the intent is not curative in nature. Surgical resection in these patients aims to reduce disease burden and increase DFS in selected patients. Both of these types of therapy are better thought of as pre-surgical therapies (5, 6). We have included the data from those receiving pseudoneoadjuvant therapy, as it helps us understand, and make informed decisions regarding surgical outcomes, safety, and response rates in those ultimately undergoing cytoreductive nephrectomy (7, 8).

## Neoadjuvant therapies

The pathology in RCC has long been considered a complex mechanism, largely driven by the deletion, mutation, or silencing of the Von Hippel Lindau (VHL) gene, a tumor suppressor gene, through either spontaneous deletion of chromosome 3p (the location of the VHL gene) or in the autosomal dominant VHL disease (9). Without its appropriate tumor suppression mechanism, an abnormal VHL gene leads to the accumulation of hypoxia-inducible factors (HIFs). This drives the production of vascular endothelial growth factor (VEGF) which leads to angiogenesis, a key pathogenic feature of RCC (10). VEGF, therefore, has been a key target for the management of RCC. To this effect, Sorafenib and Sunitinib, tyrosine kinase inhibitors (TKIs), as well as vascular endothelial growth factor receptor (VEGFR) blockers, were among the first targeted products developed for the treatment of metastatic RCC. These products were approved for advanced stage RCC by the Food and Drug Administration in 2005 and 2006, respectively, and have since been used as the control groups for many of the newer, targeted agents in subsequent trials (11, 12). Naturally, after their approval in the metastatic setting, these TKIs along with their subsequently approved counterparts were optimized and tested for use in the neoadjuvant setting. The goals of these agents in the neoadjuvant setting are different from those in the metastatic setting. Primarily, their use in the neoadjuvant setting is to optimize surgical outcomes – that is, convert unresectable tumors to resectable sizes, downsize for nephron-sparing surgery, and decrease the invasion of tumor thrombi into the inferior vena cava. Advocates of the use of these agents in the neoadjuvant setting have argued that these goals will allow for improved surgical outcomes as a result of simpler surgical processes and potentially improved long-term survival because of the removal of micrometastatic disease (5, 13). However, some experts have also challenged neoadjuvant therapy use, citing concerns about delaying definitive therapy, exposing patients to the side effects of potent VEGF inhibitors, and that these treatment options should be reserved for the advanced stage (14).

### Axitinib

Axitinib is an oral TKI that has been approved as a monotherapy and, more recently, as a combination therapy in advanced RCC (15, 16). Axitinib was first evaluated in the neoadjuvant setting by Karam et al. In 24 patients with biopsy-confirmed, nonmetastatic cT2-T3b clear cell renal cell carcinoma (ccRCC), Axitinib was administered neoadjuvantly, followed by surgical resection. The median reduction in the primary renal tumor diameter was 28.3%, with 11 patients (45.8%) showing partial response and 13 patients having stable disease according to RECIST criteria. No disease progression was observed while on Axitinib. Postoperatively, there were thirteen grade 2 and two grade 3 complications, with no grade 4 or 5 complications. It was concluded that, in patients with locally advanced non-metastatic ccRCC, Axitinib demonstrated clinical effectiveness and was generally well-tolerated when used in the neoadjuvant setting (17).

Lebacle et al. also evaluated Axitinib’s use in the neoadjuvant setting. The aim was different as it was used to downstage cT2aN0-NxM0 ccRCC patients who were ineligible for partial nephrectomy. 18 patients were enrolled. After receiving 2-6 months of neoadjuvant therapy 12 patients achieved the primary outcome, tumor size <7 cm in size, and 16 patients’ tumors decreased in diameter, and they became eligible for PN. Grade 3 adverse events were observed in 5 patients, and another 5 patients faced Clavien III-V complications after their surgeries. At the 2-year follow-up, 6 patients exhibited metastatic progression, while two experienced a recurrence. The study authors concluded that Axitinib was an appropriate neoadjuvant strategy in selected patients. Specifically, in those with cT2 ccRCC tumors, Axitinib may decrease the size of the tumor and make PN possible (18).

### Pazopanib

Like Axitinib, Pazopanib is an oral VEGFR TKI but with an expanded mechanistic range. In addition to its VEGFR inhibitive properties, Pazopanib also inhibits platelet-derived growth factor (19). Pazopanib has been approved in the metastatic setting based on its demonstrated efficacy and tolerability (20). Neoadjuvant Pazopanib was evaluated in a phase II trial. The trial evaluated the ability of neoadjuvant Pazopanib to allow for PN in localized ccRCC, similar to Lebacle et al.’s Axitinib study. Patients accepted to the trial had to meet at least one preoperative criteria: either their PN or RN was likely to yield a glomerular filtration rate of less than 30 ml/minute/1.73 m2, or their planned PN was deemed high risk due to high complexity (21), which is a R.E.N.A.L. nephrometry score of 10-12 and/or tumor proximity to the renal hilar vessels (22). The primary endpoint was tumor size reduction to allow for PN. 25 patients were enrolled in the study, and 13 were ineligible for PN based on pre-therapy assessment. After treatment, the objective tumor response rate was reached in 33% of patients, and 6 of these 13 (46%) patients became eligible for PN with the tumor diameter reduction. In addition, these patients demonstrated improved preservation of functional renal parenchyma. Notably, most patients (64%) developed grade 3 adverse events. The study noted that neoadjuvant Pazopanib reduced the size of localized ccRCC, preserving more renal tissue and making PN possible for some patients who would otherwise need a radical nephrectomy (21).

### Sorafenib

Sorafenib is an oral, multi-targeting VEGFR, platelet-derived growth factor receptor, fibroblast growth factor receptor-1, and Ras/Raf/MEK pathway inhibitor (23). After approval for treatment of RCC in the metastatic setting, Sorafenib was evaluated in the adjuvant and neoadjuvant setting. In the neoadjuvant trial, patients were randomized in a 3:1 fashion to Sorafenib or placebo (24). The primary outcomes were a reduction in tumor volume and evaluation of R.E.N.A.L scores. Ultimately, while no significant change in tumor volume was seen in the placebo arm, in the Sorafenib arm tumor volume reduction was 29%. There was no statistically significant change in R.E.N.A.L scores in the placebo, whereas it decreased in four out of nine cases within the sorafenib group.

### Cabozantinib

More recent data also suggests neoadjuvant treatment with cabozantinib contributed to tumor reduction with no disease (25). Bilen et al. demonstrated cabozantinib was clinically active and safe in the neoadjuvant setting in patients with locally advanced nonmetastatic ccRCC. 16 patients with biopsy-proven ccRCC were treated with neoadjuvant cabozantinib at a starting dose of 60 mg once per day for 12 weeks. The primary outcome of this study was an objective response rate at 12 weeks per RECIST v1.1, including complete and partial responses. Results from this analysis demonstrated that all patients had tumor reduction. 5 patients (29.4%) had a partial response (overall response rate = 0.29; 95% CI, 0.1-0.56) and 11 patients had stable disease. Progression of the disease was not observed in the patients treated with cabozantinib (25). The selected prospective studies of neoadjuvant/preoperative targeted therapy are summarized in **Table 1**.

**Table 1:** Selected prospective studies of neoadjuvant/preoperative targeted therapy.

Authors (year)	Drug	N	Dose	Duration (range)	Inclusion criteria	M1%	Histology	Median tumor diameter changes in cm	Median percentage of tumor size change	RECIST response in primary tumor (n)			RN (n)	PN (n)
										PR	SD	PD		
Cowey et al. (2010) (26)	Sorafenib	30	400 mg BID	33 days (8-59)	≥ cT2, Nany, Many	43	All (70%cc)	-0.8	-9.6%(-40 to +16)	2	28	0	30	0
Hatiboglu et al. (2017) (24)	Sorafenib	9	800 mg BID	28 days	cT1-3, N0, M0	0	All (77%cc)	-1	-29%(-61.1 to -4.9)	4	5	0	5	4
Karam et al. (2014) (17)	Axitinib	24	5 mg BID	12 weeks	cT2-3b, N0, M0	0	cc	-3.1	-28.3%(-5.3 to -42.9)	11	13	0	19	5
Lebacle et al. (2019) (18)	Axitinib	18	5-10 mg BID	60 days (58-114)	cT2a, N0-x, M0	0	cc	-1.2	-17.1%(-4.8 to -29.4)	4	13	0	1	16
Rini et al. (2015) (21)	Pazopanib	25	800 mg daily	8-16 weeks	≤pT3b, N0-x, M0	0	96%cc	-1.5	-26%(-43 to +2)	10	15	0	7	18
Powles et al. (2016) (27)	Pazopanib	104	800 mg daily	13 weeks (11-14)	M1	100	cc	-1.7	-14.4%(-21.1 to -1.4)	13	71	16	65	0
Bilen et al. (2022) (25)	Cabozantinib	16	60 mg daily	12 weeks	≥cT3, Nx or Tany, N1	0	cc		-24%(-6 to -45)	5	11	0	13	2

N, patient number; cc, clear cell type; PR, partial response; SD, stable disease; PD, progressive disease; RN, radical nephrectomy; PN, partial nephrectomy.

## Safety of anti-VEGF agents pre-operatively

A major issue with neoadjuvant therapies is the potential delay in curative surgical strategies or excessive surgical complications because of the side effects. TKIs, in general, can be difficult to tolerate and they are commonly reported issues like fatigue, stomatitis, diarrhea, rash, hand-foot syndrome, and anorexia. Additionally, because of their anti-angiogenic properties, there is specific concern regarding surgical complications like impaired wound healing (28).

In general, the concerns described above seem largely unsupported by the prospective neoadjuvant trials. No grade 4-5 adverse events and no treatment-related deaths were noted. Approximately 30-65% of patients experienced grade 3-4 adverse events like transaminitis, hypertension, gastrointestinal issues, and oral mucositis. These typically resolved with temporary discontinuation or dose reduction of the agent and, notably, there was no report of surgical delay because of these adverse events (6). Grade 1-2 adverse events were mostly comprised of TKI-specific adverse events including hand- foot syndrome and oral mucositis.

Post-operatively, 3 of the 4 published studies reported the data on complications. Importantly, chylous ascites and superficial wound dehiscence were noted particularly in the studies by Karam et al. and Rini et al. (17, 29) However, without control arms and given the small cohort sizes in the studies, it is difficult to understand the causality of these adverse events. The preference for PN over radical nephrectomies, driven by the outcome measures in these studies, may have contributed to these adverse events.

## Effect of neoadjuvant therapy on inferior vena cava thrombus

Direct extension of the RCC tumor bed into adjacent venous structures is estimated to occur in up to 10% of cases (30). When identified, tumor thrombi indicate the presence of micrometastases at the time of surgery and are associated with poorer prognoses (31). The current standard of care for this population involves complex surgical intervention, which typically includes radical nephrectomy followed by tumor thrombectomy. Larger tumor thrombi also increase the risk of perioperative and postoperative complications (32). Given these challenges, including poorer survival rates and the complexity of surgeries, there has been growing interest in exploring the benefits of neoadjuvant treatments. These treatments aim to reduce the size of tumor thrombi, thereby facilitating more favorable surgical conditions, addressing micrometastatic disease, and ultimately, enhancing the prospects for overall survival outcomes.

Data guiding the use of neoadjuvant systemic therapy for the management of tumor thrombi in RCC have predominantly come from smaller, retrospective analyses, which have produced conflicting results. Earlier datasets raised questions about the clinical utility of neoadjuvant systemic therapy in reducing the size of tumor thrombi.

Specifically, retrospective analyses published in 2011 and 2014 concluded that neoadjuvant therapy had little to no significant impact on tumor thrombi in a clinically meaningful manner (33, 34). However, it should be noted that the patients in these studies were treated with therapies that are now considered outdated and have largely been phased out of clinical use, such as Sunitinib, Bevacizumab, Temsirolimus, and Sorafenib. In a larger retrospective cohort study conducted in 2019 with 53 patients, the impact of preoperative sunitinib on patients with RCC was examined compared to those who underwent surgery directly without neoadjuvant systemic therapy (35). Among the 19 patients who received systemic therapy prior to surgery, IVC tumor thrombi were downstaged in 8 patients (42.1%) and remained stable in 10 patients (52.6%) with a median thrombus size reduction of 1.3 cm. Most notably, the authors found a statistically significant improvement in cancer-specific survival (OR 3.25, p = 0.021) through multivariate analysis and observed longer median cancer-specific survival in Kaplan-Meier analysis (72 versus 38 months, p=0.023) among patients who received neoadjuvant Sunitinib. Additionally, there was a significant reduction in perioperative blood loss in those who received neoadjuvant Sunitinib. However, it is important to note that the study’s analysis included both M0 and M1 patients, and the observed survival differences were, in part, attributed to the M1 patients, who generally have poorer outcomes. Therefore, these data should be interpreted with this context in mind.

Data from retrospective study populations often have limited clinical value and do not lead to changes in clinical practice paradigms. Considering the mixed outcomes observed in the previously mentioned retrospective studies, the NAXIVA trial was specifically designed to prospectively evaluate the therapeutic value of neoadjuvant Axitinib, focusing on its efficacy in the reduction of venous tumor thrombus extent (36). As a single-arm, multi-center, phase 2 study, the authors enrolled 20 patients with resectable RCC and venous tumor thrombus (VTT) to receive up to 8 weeks of neoadjuvant Axitinib. 35% (7 out of 20) of the patients with VTT experienced a reduction in their tumor thrombus size following treatment with Axitinib. Notably, no patients experienced an increase in VTT size. 41.2% (7 out of 17) of the patients who underwent surgery had less invasive surgery than originally planned. The authors concluded that the NAXIVA trial provided the first prospective evidence that Axitinib downstaged VTT in a significant proportion of patients which led to a reduction in the extent of surgery. A key limitation of the trial was that Axitinib is used in combination with immunotherapy in the first-line metastatic setting and only used as a single agent in subsequent lines of treatment. This presents sequencing dilemmas and challenges for patients who might progress after receiving Axitinib in the neoadjuvant setting. Given the more robust responses observed with IO+TKI therapy combinations, future prospective trials should evaluate these combinations in patients with locally advanced RCC with VTT.

### Adjuvant therapies

The adjuvant treatment strategies have been evolving with a particular focus on novel IO therapies. The standard of care for patients with localized and locoregional diseases involves definitive surgical strategies, with 5-year overall survival rates of 93% and 71% for these groups. However, the approval of Pembrolizumab in selected high-risk patients has led to a shift in treatment strategies and sparked the investigation of alternative options within this context (37).

### Patient selection

For patients with localized or locoregional disease who have undergone surgical resection, the question of which patients would benefit from adjuvant therapy is key. Most trials evaluated and stratified patients’ risk according to tumor-node-metastasis (TNM) staging. The risk of recurrence can also be calculated using either the University of California, Los Angeles (UCLA) Integrated Staging System (UISS) or the Mayo Clinic Leibovich prognostic model (38, 39). Those patients with a higher estimated risk of recurrence at five years (≥30 percent) were also likely to benefit from adjuvant therapy. However, clinically and practically, most clinicians still use TNM staging to determine those at the highest risk of recurrence and, thus, most likely to benefit from adjuvant therapy.

### IFNα and IL-2

Historical trials now provide important insight into agents that are not beneficial and, in some cases, may be detrimental. Interferon-alpha (IFN- α) and interleukin-2 (IL-2) were both studied in the 2000s as adjuvant agents. A study of high-dose IL-2 given after surgical resection in individuals with completely resected, locally advanced (T3b-4 or N1-3) or metastatic (M1) disease was found to be ineffective (40). DFS for the IL-2 and placebo groups were the same so the study was terminated early. IFN- α was also evaluated as a possible therapeutic agent. Those patients with T3-4a and/or N, M0 disease after complete surgical resection were tested with IFN- α against standard-of- care observation (29). Median OS was 7.4 years in the observation arm and 5.1 years in the treatment arm, indicating a lack of clinical benefit. POLAR-01 evaluated the combination of IFN α and low-dose IL-2 therapies in completely resected pT2-3bM0 and pN0-3M0 disease. The results were similar to those of IL-2 and IFN- α as monotherapy. Relapse-free survival (RFS) and OS were similar for the treatment and observation arms (41). Based on this data, it is clear that there is no clinical benefit to using IFN-α or IL-2, either as monotherapy or in combination in the adjuvant setting.

### VEGFR tyrosine kinase inhibitors

Sunitinib is currently approved by the Food and Drug Administration (FDA) for adjuvant therapy based on improved DFS in patients with high-risk diseases. However, it did not demonstrate a benefit in OS in any subgroup and is associated with significant toxicity. As a result, it is not a treatment that is widely used in clinical practice (42). In the S- TRAC trial, patients with locoregional, non-metastatic RCC (T3/4 and N0/x or any T stage with local node involvement and clear cell histology) were randomized in a 1:1 ratio to receive Sunitinib or placebo for 1 year. The primary endpoint of DFS was 6.8 years in the Sunitinib arm and 5.6 years in the placebo arm. Based on these data the FDA approved Sunitinib for use in the adjuvant setting for RCC patients with high-risk disease. At the conclusion of the study, OS data were immature and the number of deaths among treatment arms was equal. Upon closer analysis, it became evident that the administration came at a substantial cost. Only 56% were able to complete the full 1-year treatment, indicating that the discontinuation rate was unacceptably high. Later analyses evaluating the OS rate for Sunitinib failed to demonstrate meaningful benefit. The hazard ratio (HR) for Sunitinib vs placebo was 0.92 (95% CI 0.66 – 1.28, p = 0.6). While it technically remains approved, it is not the preferred choice in clinical practice.

Another study, the ASSURE trial, evaluated Sunitinib and Sorafenib, another VEGFR TKI, against placebo in a 1:1:1 ratio comparison (43). A total of 1943 patients with completely resected intermediate-, high-, or very high-risk RCC were randomly assigned to receive Sunitinib, Sorafenib, or placebo for up to one year. The median follow-up time was 5.8 years. Similar DFS was observed for the treatment arms: sunitinib versus placebo, with a median of 5.8 years versus 6.6 years (HR 1.02, 97.5% CI 0.85-1.23); sorafenib versus placebo, median 6.1 versus 6.6 years (HR 0.97, 97.5% CI 0.8-1.17). OS was also calculated and was similar between groups: sunitinib versus placebo (HR 1.17, 97.5% CI 0.9-1.52); sorafenib versus placebo (HR 0.98, 97.5% CI 0.75-1.28). Interestingly, in a *post-hoc* subgroup analysis, older women also experienced increased mortality. The treatment was also associated with substantial toxicity. Both VEGF TKIs were associated with hypertension, hand-foot syndrome, rash, and fatigue at significant rates (sunitinib and sorafenib vs placebo - hypertension 17% and 16% versus 4%, hand-foot syndrome 15% and 33% versus 1%, rash 2% and 15% versus <1%, and fatigue 18% and 7% versus 3% (44).

Pazopanib, another VEGFR TKI, was evaluated in the PROTECT trial, a study that compared adjuvant Pazopanib to placebo (45). Similar to other trials, and the general theme of VEGFR TKI vs placebo, DFS was similar between pazopanib and placebo (HR 0.86, 95% CI 0.70–1.06, p = 0.165). Interestingly, the study underwent a change from the initial protocol with a dose reduction amendment from 800mg to 600mg. A later analysis did not demonstrate an OS benefit (HR 1.0, 95% CI 0.80–1.26, p > 0.9). Notably, DFS was improved for those assigned to the higher daily dosing of 800mg/day (66% versus 56%, HR 0.66, 95% CI 0.49-0.9). The discontinuation rate was significantly higher in the Pazopanib group at 36% (46).

The ATLAS trial compared Axitinib against placebo in 724 patients who had undergone nephrectomy for a minimum of 1 year, up to 3 years. Axitinib failed to demonstrate a DFS benefit. As a result, in a pre-planned interim analysis, the trial was stopped early due to futility (HR 0.870, 95% CI 0.660–1.147, p = 0.3211). Axitinib was also associated with a higher rate of grade 3 toxicities (61% vs 30%) (47).

In another phase III trial, SORCE, adjuvant Sorafenib was evaluated in 1711 patients who had completely resected RCC and were at intermediate or high risk of disease recurrence. The trial had three arms: patients were randomized to either 3 years of placebo, 1 year of Sorafenib followed by 2 years of placebo, or 3 years of Sorafenib. Sorafenib did not improve DFS (HR 1.01, 95% CI 0.82 to 1.23, p = 0.946) or OS and was associated with higher rates of toxicity compared to placebo (48).

The ARISER trial studied 864 patients and was a randomized, double-blind, placebo- controlled phase 3 clinical trial that evaluated the safety and efficacy of adjuvant Girentuximab (an antibody targeting carbonic anhydrase IX) on DFS and OS (49). Girentuximab did not show statistically significant benefits in DFS (HR 0.97, 95% CI, 0.79-1.18) or OS (HR 0.99, 95% CI, 0.74-1.32). Everolimus (an mTOR inhibitor) was evaluated in the phase III EVEREST trial with resected non-metastatic RCC. The very high-risk population was defined by tumor stage 3a with grade 3 or 4; tumor stage 3b, 3c, or 4 with any grade; or nodal metastases with any tumor stage and any grade.

Everolimus failed to improve recurrence-free survival (RFS) or OS compared with placebo. It was also associated with high rates of treatment discontinuation due to its toxicity profile. Notably, RFS was improved in a subset of the study population. Those with a very high risk of disease recurrence had improved RFS (HR 0·79, 95% CI 0·65- 0·97; p=0·022), suggesting possible efficacy in this particular patient population. This finding was not replicated in the intermediate-high-risk group (HR 0·99, 95% CI 0·73- 1·35; p=0·96) (50).

Finally, several surveillance protocols have been proposed for the patients who were observed after definitive treatment and did not undergo adjuvant therapy. The American Urologic Association (AUA) and the National Comprehensive Cancer Network (NCCN) both have distinct but similar protocols (51, 52). Both of these surveillance protocols utilize a risk-stratified approach for monitoring patients and are equally effective in detecting both locoregional and distant metastases, serving as a useful tool for patient management. Ultimately, both guidelines provide an estimate of the risk of recurrence based on the extent of the disease (51, 52).

### Immune checkpoint inhibitors

IO has proven its effectiveness in the metastatic setting of RCC. The FDA has approved multiple IO combinations, including Pembrolizumab + Lenvatinib, Pembrolizumab + Axitinib, Nivolumab + Ipilimumab, and Avelumab + Axitinib, all of which were approved for use in the first line setting for metastatic RCC. Considering the established use of IO in metastatic RCC and its growing clinical significance in other genitourinary (GU) and non-GU cancers, it is reasonable to question its potential role in the perioperative RCC space.

Currently, only Pembrolizumab is FDA-approved as adjuvant therapy for RCC patients at high risk of recurrence after nephrectomy. In the Keynote-564 study, which was a double-blind, placebo-controlled phase III trial, patients with histologically confirmed RCC treated with nephrectomy were randomized to receive either Pembrolizumab every three weeks for up to 1 year (17 cycles in total) or placebo. The risk for disease recurrence was defined as intermediate to high risk (pT2 tumors with grade 4 or sarcomatoid features N0, M0; or pT3, any grade, No, M0), high risk (pT4, any grade, N0, M0; or any pT, any grade, N1, M0), or M1 with no evidence of disease (resection of all oligometastatic sites (M1) with no evidence of disease (NED) within one year of nephrectomy). At the median follow-up duration of 24 months, Pembrolizumab improved DFS compared with placebo across all disease risk groups (24-months DFS 77.3% versus 68.1%, HR 0.68, 95% CI 0.53-0.87). This treatment also demonstrated an OS benefit across all disease risk groups (24-month OS 96.6% (%95 CI, 94.3-98) versus 93.5% (95% CI, 90.5-95.6)). However, OS data are not statistically significant yet and longer follow-up is required for the data maturation. Notably, the PD-L1 combined positive score (CPS) was greater than or equal to 1 for 73.6% of patients on Pembrolizumab and 76.9% on placebo. The discontinuation rate was significantly higher in the treatment arm at 20.7% compared to 2.0% in the placebo arm. Based on these data, the FDA and the European Medicines Agency (EMA) approved Pembrolizumab for adjuvant treatment of RCC patients at high risk of recurrence following nephrectomy. Interestingly, the study also included 58 patients with oligometastatic disease that was resected with NED within one year of nephrectomy (M1, NED). In this group of patients, adjuvant Pembrolizumab also increased DFS compared to placebo (median not reached versus 12 months, HR 0.28, 95% CI 0.12-0.66). Based on this data, the FDA also granted approval for Pembrolizumab’s use following nephrectomy and resection of metastatic lesions (53).

**Figure 1:**
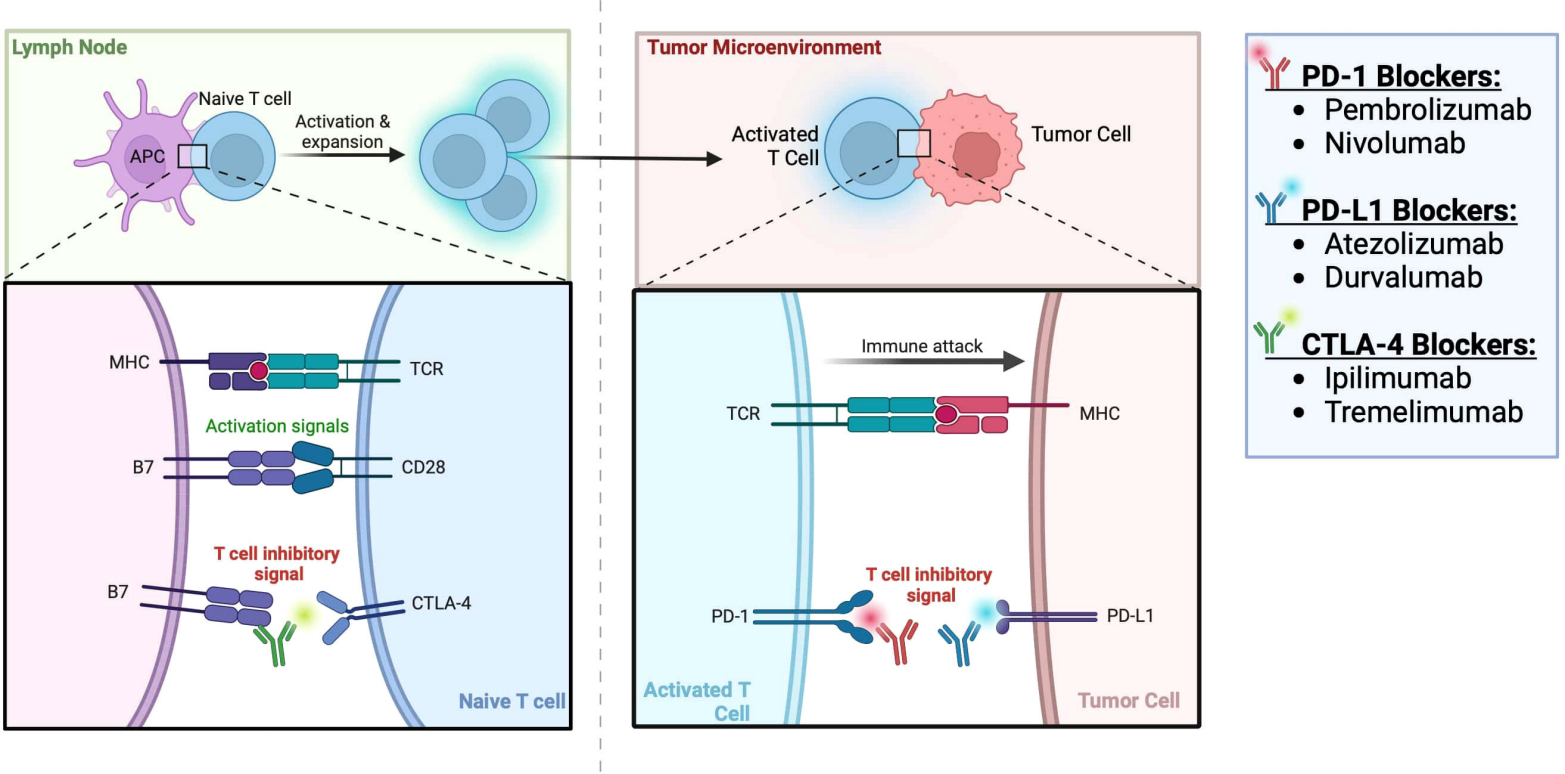
Immune checkpoint inhibitors for RCC (56).

**Figure 2:**
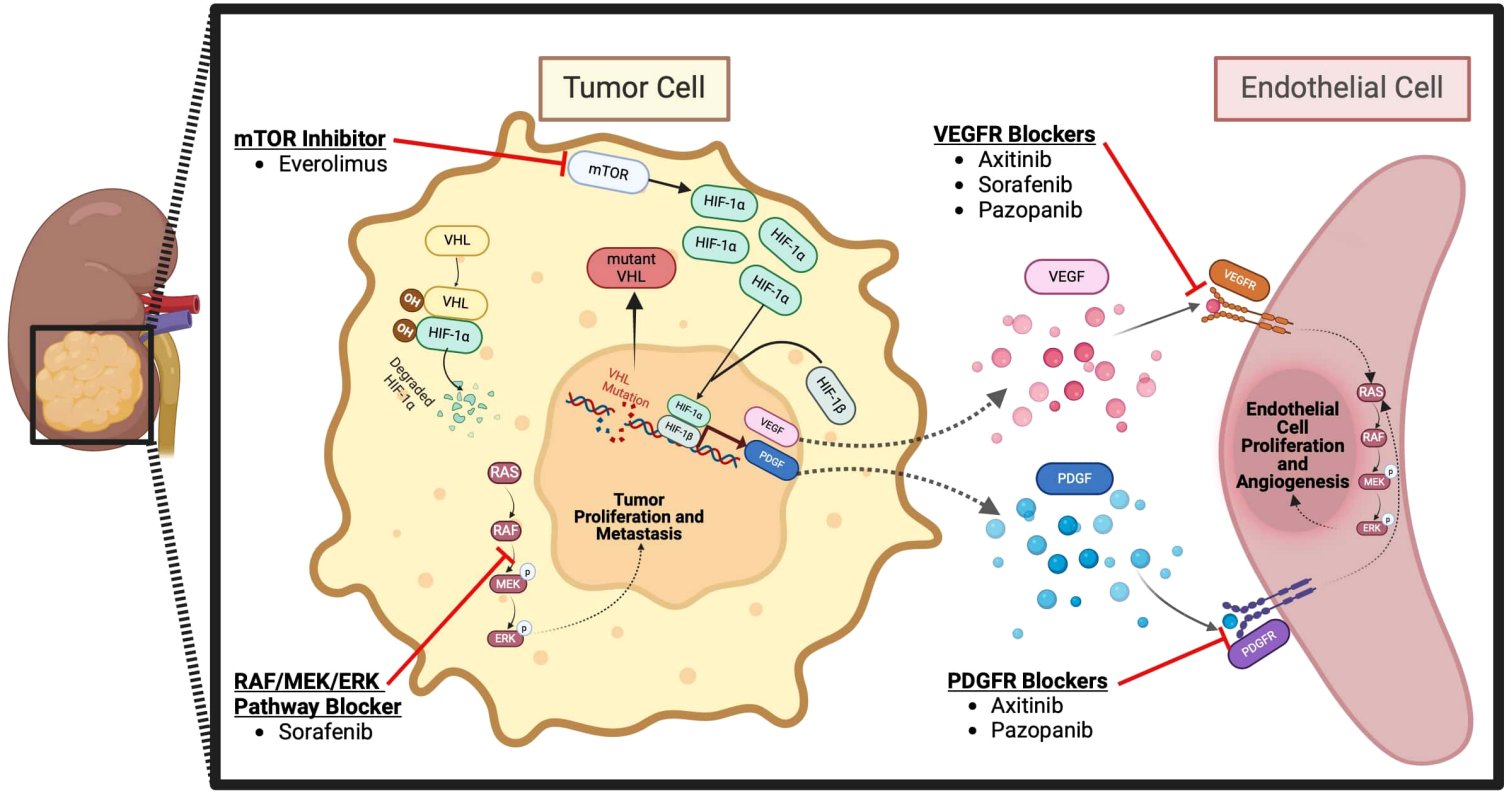
Targeted neoadjuvant therapies for RCC (57).

Some trials have also evaluated the use of IO therapy in the neoadjuvant setting. The Neoavax, a phase II study, assessed neoadjuvant Avelumab and Axitinib before nephrectomy in 40 patients with cT1b–4cN0–1M0, grades 3–4 RCC. None of the patients on trial had tumor progression while 30% (12 patients) had a partial response to the treatment (54). Neoadjuvant nivolumab was also evaluated in an early phase small study of 17 patients with nonmetastatic high-risk RCC. In this study, all 17 patients had stable disease according to the RECIST criteria, and the drug was found to be safe and well tolerated. 2 patients (11.8%) experienced grade 3 events. 10 patients (58.8%) experienced adverse events of any grade potentially attributable to nivolumab (all were grade 1-2), and there were no grade 4-5 adverse events (55). The mechanism of action of ICIs for RCC is shown in **Figure 1** and the targated neoadjuvant therapies for RCC is schematized in **Figure 2**.

### Recently reported and current trials

There are a number of trials ongoing at the time of this publication and several others that have recently reported evaluating the efficacy and safety of adjuvant therapy in RCC. PROSPER RCC study evaluated the patients with clinical stage T2Nx, N+ any T, or M1 patients with planned resection. All patients in the study were planned to be definitively treated so that they would effectively be considered M1 NED. The patients with high-risk RCC were randomly assigned in an open-label design to undergo surgery alone, or an approach of priming the immune system with nivolumab prior to full or partial removal of the kidney followed by additional nivolumab. In the intervention arm, patients received one dose of nivolumab prior to surgery followed by adjuvant nivolumab every 4 weeks for up to 9 cycles. Notably, this approach was strongly supported by the patient advocates. However, this trial was stopped early because, at a planned interim analysis, it showed no difference (HR 0.97, 95% CI 0.74 – 1.28; p 1-sided = 0.43) in RFS between the arms in a patient population that included both clear cell and non–clear cell disease subtypes (58).

RAMPART is an ongoing multi-arm multi-stage (MAMS) platform trial in those patients with resected locally advanced RCC (both clear cell and non-clear cell histological subtypes are included in the study), with no residual macroscopic disease, who are at high or intermediate risk of relapse (Leibovich score 3-11). The trial is assessing if Durvalumab monotherapy or the combination of Durvalumab and Tremelimumab (an inhibitory agent against cytotoxic T-lymphocyte-associated protein 4 [CTLA-4]) can improve DFS or OS compared to the active monitoring. Participants are randomly assigned (3:2:2) to Arm A - active monitoring (no placebo) for one year; Arm B - Durvalumab for one year; or Arm C - combination therapy with Durvalumab for 1 year plus two doses of Tremelimumab for the first two cycles. The primary outcomes will be measured as DFS and OS, and secondary outcomes will be measured for safety, metastasis-free survival, RCC specific survival, quality of life, and patient and clinician preferences (59, 60).

Checkmate-914 study enrolled patients with localized ccRCC who were at high risk of relapse after radical or partial nephrectomy between 4–12 weeks before random assignment (61, 62). In part A of the study, the patients were randomized to receive nivolumab plus ipilimumab or placebo, and the results were reported in early 2023.

Adjuvant therapy with Nivolumab plus Ipilimumab 5-year DFS Kaplan-Meier estimates 109.3 months (95% CI 83.9-134.6 months). Part B of the study, which is currently underway, evaluates the combination mentioned above versus single agent nivolumab for 1 year. DFS is the primary endpoint with safety serving as the secondary endpoint (61).

IMmotion010 was a randomized, double-blinded, multicenter, phase 3 trial that evaluated Atezolizumab in the adjuvant setting. After nephrectomy with or without metastasectomy, patients were randomly assigned in a 1:1 ratio to receive Atezolizumab or placebo once every 3 weeks for 16 cycles or 1 year. The primary endpoint was DFS. The results were reported in late 2022, revealing a median investigator-assessed disease-free survival of 57.2 months (95% CI 44.6 to not evaluable) with Atezolizumab and 49.5 months (47.4 to not evaluable) with placebo (HR 0.93, 95% CI 0.75-1.15, p=0.50). The study investigators found Atezolizumab as adjuvant therapy increased the risk of recurrence and showed no evidence of improved clinical outcomes compared to placebo. As a result, the study sponsor decided to terminate the study before the protocol-defined end-of-study (63).

LITESPARK-022 is a recently opened study that is currently ongoing, actively recruiting patients. It evaluates the combination of oral Belzutifan (a HIF-2 alpha inhibitor) plus Pembrolizumab, compared to placebo plus Pembrolizumab in the adjuvant treatment of RCC patients after nephrectomy. Patients with intermediate-high risk (pT2, Grade 4 or sarcomatoid, N0, M0; pT3, any grade, N0, M0), high risk (pT4, any Grade N0, M0; pT any stage, any grade, N1, M0), or M1 NED are actively being recruited. Patients must have undergone nephrectomy and/or metastasectomy ≤12 weeks before randomization and be tumor free, confirmed by imaging. Patients will be randomly assigned in a 1:1 ratio to receive either Belzutifan orally once daily plus Pembrolizumab every 6 weeks or oral placebo plus Pembrolizumab. Pembrolizumab treatment will be administered for up to 9 doses (ã1 year); Belzutifan and placebo may be continued for a maximum of 54 weeks. The primary endpoint of the study is DFS, and secondary outcomes include OS and safety (64).

Selected prospective studies of adjuvant/perioperative therapy are summarized in **Table 2**. Additionally, other relevant ongoing neoadjuvant/perioperative trials are shown in **Table 3**.

**Table 2:** Selected prospective studies of adjuvant/perioperative therapy.

Trial Name, Clinical Trials NCT identifier	Inclusion Criteria	Treatment	Primary Endpoint	Start Date	Planned Completion
**Keynote-564** **(NCT03142334)** (53)	Intermediate-/high-risk RCC: o pT2, G4 or sarcomatoid,N0,M0 o ≥ pT3, Gany, N0, M0 High-risk RCC: o Tany, Gany, N1, M0 o M1 with NED	Pembrolizumab (1 year)	DFS at 24 months 77.3% vs. 68.1%in placebo (HR 0.68; 95% CI 0.53 to 0.87, p=0.002).	June 2017	December 2020
**IMmotion010** **(NCT03024996)** (63)	o pT2 G4, N0,M0 o pT3a G3-4, N0, M0 o pT3b/c or pT4, Gany, N0, M0 o pTx, N1, M0 o M1 with NED	Atezolizumab (1 year)	DFS was 57.2 months (95% CI 44.6 to not evaluable) with atezolizumab and 49.5 months (47.4 to not evaluable) with placebo (HR 0.93, 95% CI 0.75–1.15, p=0.50)	January 2017	May 2022
**PROSPER** **(NCT03055013)** (58)	o ≥ cT2, Nx, M0 o cTany, N1,M0 M1 with planned resection	Nivolumab q4weeks with one dose given preoperatively followed by 9 adjuvant dose	Intervention group did not significantly improve RFS compared with surgery alone (HR, 0.97, 95% CI 0.74-1.28, 1-sided p=0.43)	February 2017	March 2022
**RAMPART** **(NCT03288532)** (59, 60)	o ≥Leibovich score 3 o All patients at high risk are included first. Following that, intermediate-risk patients are randomly selected until they make up 25% of the cohort	Durvalumab or Durvalumab plus Tremelimumab (1 year)	DFS and OS	July 2018	July 2024
**LITESPARK-022** **(NCT05239728)** (64)	Intermediate-high risk o pT2, G4 or sarcomatoid, N0, M0 o pT3, Gany, N0, M0 High risk RCC o pT4, Gany, N0, M0 o pTany, Gany, N1, M0 o M1 with NED	Belzutifan plus Pembrolizumab (1 year)	DFS	March 2022	October 2026
**Checkmate 914** **(NCT03138512)** (61)	o pT2a, G3-G4, N0, M0 o ≥ pT2b, G any, N0 M0 o pT any, G any, N1 M0	Nivolumab plus Ipilimumab (1 year)	5-year DFS is 109.3 months (95%CI,83.9-134.6 months)	July 2017	July 2024
**KEYNOTE-426** **(NCT02853331)** (65)	o RCC with clear cell component with or without sarcomatoid features o Locally advanced or metastatic disease	Pembrolizumab + Axitinib versus Sunitinib	PFS was 16 mo (95% CI, 14–20) with pembrolizumab plus axitinib and 11 mo (95% CI, 8.9–13) with sunitinib (HR 0.68; 95% CI, 0.58–0.80. At 36 mo, the OS rate was 63% in the combination arm and 54% in the sunitinib arm (median: 46 vs 40 mo; HR 0.73; 95% CI0.60–0.88)	September 2016	December 2025

DFS, disease-free survival; OS, overall survival; RFS, recurrence-free survival; PFS, progression-free survival; ORR, objective response rate.

**Table 3:** Selected ongoing neoadjuvant/perioperative clinical trials.

Trial Name, Clinical Trials (NCT identifier)	Trial Phase	N	Inclusion criteria	Study Design	Primary Endpoint	Start Date	Estimated Completion
**NORDIC-SUN** **(NCT03977571)** (66)	Phase-III	400	o Histologically confirmed mRCC of any histology o No prior therapy for mRCC	Nivolumab+ Ipilimumab with or without CN	Overall survival	July 2020	September 2028
**PROBE** **(NCT04510597)** (67)	Phase-III	364	o Histologically confirmed mRCC of any histology except collecting duct o Treatment naive or previously treated.	ICI-based regimens with or without CN	Overall survival	March 2021	July 2033
**Cyto-KIK** **(NCT04322955)** (68)	Phase-II	48	o Histologically confirmed mRCC with clear cell component o No prior therapy for mRCC	Cabozantinib (stopped 21 days prior to surgery)+ Nivolumab+ CN versus Cabozantinib (stopped 14 days prior to surgery)+ nivolumab+ CN	Percentage of participants with a complete response	June 2020	February 2027
**NESCIO** **(NCT05148546)** (69)	Phase-II	69(estimated)	o Histologically confirmed resectable clear cell RCC o Intermediate to high-risk patent: cT1b-cT2a, G4, N0, M0 or cT2b, G3, N0 M0 or cT3-T4 Gany,N0,cM0 or cTany, cN1(fully resectable), cM0 o No prior immunotherapy	Nivolumab versus Nivolumab + Ipilimumab versus Nivolumab + Relatlimab	Pathologic response rate	April 2022	April 2029
**WINSHIP4955-20** **(NCT04393350)** (70)	Phase-II	22(estimated)	o Biopsy proven RCC with clear cell component o cT≥3, Nx, M0 or cTany, N1 or deemed unresectable by surgeon o Previously untreated	Lenvatinib + pembrolizumab before nephrectomy	Objective response rate (CR and PR)	June 2020	August 2024
**SUNNIFORECAST** **(NCT03075423)** (71)	Phase-II	316	o Histological confirmation of non-clear cell RCC o Previously untreated o Locally advanced and unresectable o Non-metastatic	Standard of care versus Ipilimumab + Nivolumab	Overall survival	November 2020	May 2024
**PDIGREE** **(NCT03793166)** (72)	Phase-III	1175 (estimated)	o Histologically confirmation of RCC with clear cell component o Any metastatic disease o Previously untreated	Ipilimumab + Nivolumab followed by either Nivolumab versus Cabozantinib+ Nivolumab	Overall survival	June 2019	September 2024

mRCC, metastatic renal cell carcinoma; CN, cytoreductive nephrectomy; ICI, immune checkpoint inhibitor; CR, complete response; PR, partial response; SD, stable disease; PD, progressive disease.

Furthermore, there have been some phase III clinical trials that included metastatic patients without prior nephrectomy. An exploratory *post hoc* analysis from the phase III CheckMate 214 trial showed the effectiveness of Nivolumab plus Ipilimumab (NIVO+IPI) compared to Sunitinib in 108 patients with advanced RCC who haven’t had nephrectomy, a group often overlooked in clinical studies. After a minimum of four years of follow-up, results showed a significant advantage for NIVO+IPI in terms of PFS, ORR, and OS compared to Sunitinib. The respective figures were 8.1 months, 34%, and 26.1 months for NIVO+IPI, versus 11.9 months, 15%, and 14.3 months for the Sunitinib arm. Notably, 35% of NIVO+IPI patients saw a 30% reduction in their renal tumor size versus 20% of patients with Sunitinib, with safety profiles aligning with the larger study cohort. These findings suggest that NIVO+IPI can offer significant survival and tumor reduction benefits for advanced RCC patients without previous nephrectomy, highlighting its potential in a population where such treatment outcomes are largely unknown (73).

A JAVELIN Renal 101 phase III trial, compared the effect of Avelumab plus Axitinib (A+Ax) with Sunitinib therapy for advanced RCC patients. For patients without a history of nephrectomy, ORR was better in the A+Ax arm, 34.4 months versus 16.9 months (15). In addition, 34.5%of patients in A+Ax compared to 9.7% in Sunitinib experienced at least a 30% reduction in the size of renal target lesions from baseline. The median time to achieve at least a 30% reduction was 4.4 months for A+Ax and 7.1 months for Sunitinib (74).

The phase III CLEAR study demonstrated that Lenvatinib plus Pembrolizumab (L+P) significantly improved efficacy versus Sunitinib as the first-line treatment for patients with advanced RCC (75). When the patients without prior nephrectomy were analyzed, the median OS was not reached for the L+P group, and it was 30.7 months for the Sunitinib group. Additionally, the median PFS was 22.1 months for L+P, versus 7.5 months for Sunitinib, and ORRs were 71.8% and 27.0%, respectively (76). Additionally, 76.8% of patients in the L+P compared to 25.6% in the Sunitinib experienced at least a 25% reduction in the size of renal target lesions from baseline, and 21.4% of patients in the L+P group achieved a 50% or greater reduction, outperforming the 7% in the Sunitinib group (77).

The CheckMate 9ER phase III trial compared Nivolumab plus Cabozantinib (N+C) with Sunitinib for the treatment of previously untreated patients with advanced RCC. In a subgroup analysis of patients without prior nephrectomy, PFS, ORR, and OS were superior in the N+C arm, with respective values of 11.3 months, 42%, and 31.11 months, compared to 7.06 months, 23%, and 27.07 months in the Sunitinib arm. Furthermore, 45% of patients treated with N+C, compared to 30% with Sunitinib, achieved a reduction of at least 30% in the size of renal target lesions from baseline. Moreover, those receiving N+C reported an improvement in health-related quality of life compared to those on Sunitinib (78, 79).

The phase III clinical trials that included metastatic RCC patients without prior nephrectomy are reported in **Table 4**.

**Table 4:** Phase III clinical trials that included those patients without prior nephrectomy.

Authors (year)	Drug	N	PFS (months)	ORR (%)	OS (months)	% of people who acchive tumor shrinkage	RECIST response in primary tumor (%)			
							CR	PR	SD	PD
**CheckMate 214** **(NCT02231749)** (73)	ArmA: Nivolumab+Ipilimumab ArmB: Sunitinib	ArmA:53 ArmB:55	ArmA:8.1 (95% CI 5.5–21) ArmB:11.9 (95% CI 8.4–18)	ArmA:34(95% CI 22–48) ArmB:15, 95% CI 6.5–27	ArmA:26.1(95% CI 14–35) ArmB:14.3(95% CI 9.7–23)	≥30% of shrinkage ArmA: 35% ArmB: 20%	ArmA: 0 ArmB: 0	ArmA: 18 (34) ArmB: 8 (15)	ArmA: 20(38) ArmB: 28(51)	ArmA: 11(21) ArmB: 7(13)
**JAVELIN Renal 101** **(NCT02684006)** (15, 74, 80)	ArmA: Avelumab+Axitinib ArmB: Sunitinib	ArmA:90 ArmB:89	NA	ArmA:34.4 ArmB:16.9	NA	≥30% of shrinkage ArmA: 34.5% ArmB: 9.7%	NA	NA	NA	NA
**CLEAR** **(NCT02811861)** (75–77)	ArmA: Lenvatini+Pembrolizumab ArmB: Sunitinib	ArmA:78 ArmB:74	ArmA:22.1 ArmB:7.5	ArmA:71.8 ArmB:27.0	ArmA: Not reached ArmB: 30.7	≥25% of shrinkage ArmA: 76.8% ArmB: 25.6% ≥50% of shrinage ArmA: 21.4% ArmB: 7%	ArmA: 2(2.6) ArmB: 1(1.4)	Arm A: 54 (69.2) Arm B: 19 (25.7)	Arm A: 13 (16.7) Arm B: 31 (41.9)	Arm A: 4 (5.1) Arm B: 14 (18.9)
**Checkmate 9ER** **(NCT03141177)** (78, 79)	ArmA: Nivoluma+Cabozantinib ArmB: Sunitinib	ArmA:101 ArmB:95	ArmA:11.30 (95% CI 8.80–15.97) ArmB:7.06 (95% CI 5.4–9.40)	ArmA:42(95% CI 32–52) ArmB:23(95% CI 15–33)	ArmA:31.11 (95% CI 22.28-NE) ArmB:27.07 (195% CI 9.38-NE)	≥30% of shrinkage Arm A: 45% Arm B: 30%	ArmA: 5(5) ArmB: 0(0)	ArmA: 37(37) ArmB: 22(23)	ArmA: 41(41) ArmB: 42(44)	ArmA: 7(7) ArmB: 14(15)

N, patient number; PFS, progression-free survival; ORR, objective response rate; OS, overall survival; CR, complete response; PR, partial response; SD, stable disease; PD, progressive disease; NA, not available.

### Role of cytoreductive nephrectomy

While definitive surgical resection has been extensively studied in localized and locally advanced settings, its role in the metastatic setting is still debated. Surgical resection of the primary tumor can be performed for palliative purposes to manage conditions such as gross hematuria, relieve abdominal pain, or address paraneoplastic syndromes. However, the therapeutic efficacy of nephrectomy in this context is a subject of ongoing debate. In recent years, the landscape of systemic treatment for metastatic RCC has changed considerably, yet our understanding of the role of cytoreductive nephrectomy (CN) has not advanced at the same oage. Historically, CN was considered the standard of care, offering a survival benefit when performed prior to treatment with IFN- α (81). However, the adoption and standardization of targeted therapies questioned the role of CN.

CARMENA and SURTIME were two pivotal phase III trials that questioned the use of CN in the era of targeted therapy. SURTIME examined whether a period of Sunitinib therapy before CN improved outcomes compared with immediate CN followed by Sunitinib (82). Although the trial did not meet its primary endpoint for PFS with deferred CN, It did reveal a modest survival benefit for this approach. Conversely, CARMENA, a randomized control phase III noninferiority trial, evaluated the efficacy of CN followed by Sunitinib against Sunitinib monotherapy in patients with metastatic RCC. The findings indicated that Sunitinib alone was not inferior to combination of nephrectomy followed by sunitinib in patients with metastatic RCC with intermediate or poor-risk disease (8).

The use of IO combinations and the increased number of systemic therapy options has further questioned the use of CN. Keynote-426, Checkmate 9ER, and Checkmate 214 are three pivotal trials that led to the approval of IO combinations and which reflect the current standard of care. Importantly, few patients with an intact primary tumor were included in these studies (17-30%) (16, 83, 84). Recent *post hoc* analysis from the pivotal CLEAR trial demonstrated an improvement in OS, PFS, and ORR for patients with an intact primary tumor who received Lenvantinib with Pembrolizumab when compared to Sunitinib (76). While these results do not provide definitive evidence of the lack of utility of CN, they do suggest that the primary tumor may respond similarly to systemic therapies as metastatic sites do.

To better understand the role of CN in the current landscape of IO therapy, multiple prospective clinical trials have been ongoing. NORDIC-SUN and PROBE are two phase III clinical trials evaluating the clinical impact of CN in the metastatic setting. NORDIC-SUN (NCT03977571) will compare the efficacy of Nivolumab plus Ipilimumab with or without CN. In contrast, PROBE (NCT04510597) will evaluate standard of care immunotherapy-based drug combinations with surgery versus the same drug combinations without surgery. Both of these trials have OS as their primary endpoint.

Cyto-KIK (NCT04322955), an open-label phase II, multicenter clinical trial, is evaluating a combination of Nivolumab and Cabozantinib prior to CN in patients with metastatic disease, focusing on complete response as its primary endpoint. Secondary endpoints include median size reduction of the primary tumor, ORR, PFS, OS, and surgical outcomes using the Clavien-Dindo classification system.

Consideration should also be given to the inherent risks associated with surgical intervention. Recently reported data from a large retrospective analysis concluded that postoperative complications after RN were approximately 22% for any complication and 4% for major complications (85). Additionally, as systemic therapies continue to provide durable responses and patients are becoming long-term survivors of metastatic disease, issues with nephrectomy leading to chronic kidney disease (CKD) are becoming more evident. Recent data estimated postoperative stage ≥3b CKD can range from 21% to as high as 69% depending on the patient’s age, diabetes status, and preoperative kidney function (86). While CN previously had a clearer role in the metastatic setting, its current use in the landscape of front-line IO-based systemic therapy needs through evaluation in prospective trials.

## Discussion

In this review, we examined the use of neoadjuvant and adjuvant therapies in the setting of localized RCC, an area that is rapidly evolving. In the neoadjuvant setting, the benefits of the treatment in combination with surgery remain questionable. These agents have demonstrated clear benefits in the metastatic setting and, in theory, should also show benefits in the localized setting. However, this is not currently the case. While theoretical benefits do exist, the practical disadvantages of neoadjuvant therapy make it unsuitable for widespread usage in the current treatment approach for localized RCC. Systematic analyses have revealed a clear lack of DFS or OS improvement (87, 88). Further investigation needs to be conducted to determine the ideal treatment regimen and duration as well as the specific patient population that would benefit most from therapy.

As discussed above, most trials evaluating neoadjuvant therapy employed single-agent VEGFR TKI use. Several issues prevent the adoption of these agents in the neoadjuvant setting. Modest benefits in objective outcomes, combined with vast heterogeneity in the trial design contribute to the lack of acceptance of VEGFR TKI in the neoadjuvant setting (17, 18, 20, 21). Additionally, differences in the optimal timing and administration of the VEGFR TKI led to a lack of standardization in the interpretation of the results of those trials. These trials also evaluated a small number of patients, and except for one study, none had a control study arm. Apart from one study, long-term survival and oncologic outcomes were not reported as well as the primary outcome of interest was tumor response rate and immediate surgical outcome (ability to perform partial nephrectomy following neoadjuvant therapy). In addition, the side effect profile of these agents also needs to be considered. While the TKI adverse events were considered acceptable by the study authors, the impact of these TKIs to operative complications is difficult to discern. Also, the absence of a control arm in most studies prevented true calculation of the effect of TKI on the emergence of operative complications.

The ongoing trials have shifted focus from the use of single-agent TKIs to combination therapy with IO + TKI use in the neoadjuvant setting. Importantly, the outcomes being examined have also changed. Perioperative outcomes are no longer the primary interest. Instead, oncologic outcomes such as DFS, PFS, and OS are being examined, and radiological and pathological response rates are being studied to determine the immediacy of the therapy’s effect. These trials utilize a similar schema to the metastatic setting but are employing fewer cycles and shorter treatment periods (4-12 weeks of neoadjuvant treatment followed by surgical resection). Most of these trials evaluating IO+TKI therapy also include IO therapy in the adjuvant setting to create a perioperative trial period rather than a purely neoadjuvant design.

Recent trials are also studying the use of IO + TKI + HIF inhibitors and TKI + HIF inhibitors in the perioperative setting, marking an exciting development that builds on the experience of HIF inhibitors in those patients with VHL mutations causing benign and malignant neoplasms (89). Another ongoing development is the use of stereotactic ablative radiotherapy (SAbR) in the neoadjuvant setting. An ongoing phase II trial (NCT02473536) that evaluates the use of SAbR in the neoadjuvant setting builds on the success of its use in a single-arm phase 1/2 prospective trial where it demonstrated objective success in RCC patients with inferior vena cava thrombus (90, 91). Also, neoadjuvant SAbR therapy with or without systemic therapy is under evaluation. Specifically, one ongoing trial (NCT05024318) investigates SAbR alone or in combination with Pembrolizumab followed by nephrectomy (92).

The adjuvant landscape in early stage RCC is also undergoing changes. As discussed earlier, Sunitinib offers modest benefits at the expense of significant toxicity. The current treatment paradigm suggests Pembrolizumab is the most appropriate adjuvant treatment for selected high-risk patients. Though the definition of high risk in Keynote- 564 varies considerably (pT2 to M1 patients who underwent resection have no evidence of disease), and the most significant benefit is seen in those with the highest recurrence risk of disease. M1 patients had considerably more favorable results (HR 0.29, 95% CI 0.12–0.69) as opposed to M0 patients (HR 0.74, 95% CI 0.57–0.96), as seen in the subgroup analysis. This isolated agent has demonstrated positive results and offers a key benefit in the highest risk patients, highlighting the importance of patient selection.

While biomarker development is ongoing in RCC, it does not yet have an established clinical role. Therefore, patient selection effectively relies on sound clinical judgement and appropriate and effective interpretation of TNM staging. Sequencing of therapies also remains an important concept to understand in the adjuvant setting. Recent data from the CONTACT-03 trial has demonstrated a lack of benefit with continuing IO in patients who have been previously exposed to IO. This suggests that using IO after progression on prior IO should not be the standard of care (93).Therefore, there is no ideal treatment sequencing strategy in those who received IO in the adjuvant setting and progress to a metastatic process. The results of CONTACT-03 will likely change the treatment paradigm of IO-treated adjuvant patients who progress to metastatic disease. It is worth noting that these adjuvant trials studied DFS as a primary endpoint, and OS data will be reported once mature. Several ongoing trials are still evaluating the role of IO therapies and IO + HIF inhibitors in the adjuvant setting (59, 94). The conclusion of these trials will further shape our treatment sequencing and paradigms in the years to come.

## Conclusion

While the treatment strategies in both the neoadjuvant and adjuvant setting have evolved, the recent trial data has explored the use of IO + TKI in the perioperative period in high-risk groups, as opposed to absolute neoadjuvant and adjuvant treatments. That is, clinical treatment strategies are shifting to IO + TKI in the neoadjuvant setting followed by surgical resection and then IO treatment in the adjuvant setting. Clinical outcomes are being evaluated with greater interest along with surgical outcomes, and data from several large randomized clinical trials in the perioperative space are pending.

Certainly, our treatment paradigms will shift but patient selection, timing, agent selection, and other clinical factors are yet to be clarified. We hope, with the conclusion of the pending trials, that our perioperative treatment strategy will be clearer and patient selection for more aggressive treatment options will be optimized. Additionally, exploratory biomarker evaluation and correlative data from these studies will undoubtedly shed light on important molecular features of RCC which will help us to understand the disease biology and processes driving RCC. With the aggressive expansion of combination therapy in the metastatic setting, we hope and expect that the current standard of care in the perioperative space will be challenged to provide optimal outcomes for our patients against this challenging disease.

## Author contributions

RG: Writing – review & editing, Writing – original draft, Investigation, Data curation, Conceptualization. EK: Writing – review & editing. VM: Writing – review & editing, Supervision. MB: Writing – review & editing, Supervision.
